# Circadian characteristics of term and preterm labors

**DOI:** 10.1038/s41598-024-54490-2

**Published:** 2024-02-18

**Authors:** Miha Moškon, Urša Kovač, Lucija Raspor Dall’Olio, Ksenija Geršak, Gorazd Kavšek, Eva Bojc Šmid, Andreja Trojner Bregar, Damjana Rozman

**Affiliations:** 1 Faculty of Computer and Information Science, University of Ljubljana, Ljubljana, Slovenia; 2 Centre for Functional Genomics and Bio-Chips, Institute for Biochemistry and Molecular Genetics, Faculty of Medicine, University of Ljubljana, Ljubljana, Slovenia; 3 Research Unit, Division of Gynaecology and Obstetrics, University Medical Centre Ljubljana, Ljubljana, Slovenia; 4 Department of Gynaecology and Obstetrics, Faculty of Medicine, University of Ljubljana, Ljubljana, Slovenia

**Keywords:** Preterm birth, Spontaneous birth, Circadian rhythms, Cosinor model, Computational biology and bioinformatics, Developmental biology, Molecular biology, Systems biology, Endocrinology

## Abstract

The labor is a physiological event considered to have its own circadian (diurnal) rhythm, but some of the data remain conflicting, especially for preterm births. In this retrospective study, we analyzed the circadian trends of labor onset times in the Slovenian birth cohort from 1990 to 2018 with over 550,000 cases of singleton births. The number of term and preterm labor onsets was calculated for each hour in a day and circadian trends were evaluated for each of the study groups by modeling with a generalized Poisson distribution linked with the cosinor regression model using logarithmic link function. The induced labors were taken as the control group since the timing of labor depends mostly on the working schedule of personnel and not on the intrinsic rhythmic characteristics. For induced labors, the main peak in the number of labor cases was observed in the late morning hours (around 10 AM) for all gestational ages. The prominence of this peak becomes smaller in spontaneous premature labors with gradually disrupting rhythmicity in very preterm and extremely preterm cases. Labors starting with spontaneous contractions peak between 6 and 7 AM and lose the rhythmicity at 35 weeks of gestation while labors starting with a spontaneous rupture of membranes peak at 1 AM and lose the rhythmicity at 31 weeks of gestation, suggesting differences in underlying mechanisms. According to our knowledge, this is the first study that shows differences of circadian trends between different types of spontaneous labors, i.e., labors initiated with contraction and labors initiated with a spontaneous rupture of membranes. Moreover, the obtained results represent evidence of gradual disruption of rhythmicity from mild to extreme prematurity.

## Introduction

Biological processes are often periodic or rhythmic, with a spectrum of frequencies, ranging from milliseconds to years. The most common of these rhythms are those with a periodicity close to 24 h, namely, the circadian rhythms^1^. Circadian rhythms are evolutionarily conserved biological mechanisms of all organisms and allow adaptation to cyclic changes in the environment. They are universal in the living systems and drive multiple processes of our bodies that include metabolism, hormone secretion, regulation of body temperature, etc. Such rhythmic behavior is generated by biochemical oscillators composed of rhythmically expressed proteins of the core clock, the activators CLOCK and BMAL1, and the repressors of the PER and CRY families, that are expressed in anti-phase^2–5^. The inner clock in the brain represents the central oscillator in the suprachiasmatic nucleus (SCN) of the anterior hypothalamus. The central oscillator has two major roles. Firstly, it serves to coordinate internal time with the external world, to temporally optimize behavior and physiology, and secondly, to coordinate the peripheral oscillators that reside in other tissues and must be maintained in a synchrony^2,6,7^. Apart from that, organs possess varying sets of clock-controlled genes with tissue-specific phase distributions^8^ that fit with their physiological roles.

The well-coordinated circadian system governs almost all biological processes that include a sleep–wake cycle, body temperature, feeding, reproduction, hormone secretion, glucose homeostasis, cell-cycle regulation, etc. Recent studies show also mutual interactions between the rhythms of the host and the commensal symbiotic microbes^9^, which can represent beneficial or harmful interactions. Importantly, when the inner clock is long-term^10^ misaligned with the environmental cues, this may lead to pathologies, including different types of cancer and obesity with metabolic syndrome^11^.

Among the normal physiological events under the clock control is labor which is believed to have its own circadian rhythm^12^. A growing body of evidence demonstrates the long-term impact of the pregnancy environment on the development of the fetus and circadian system's relation to pregnancy and infant growth^13^.

This study aims to bridge the gap between circadian rhythms and pregnancy outcomes, leveraging a large retrospective dataset from the Slovenian birth cohort. By examining the rhythmic behavior of preterm births initiated by contractions or spontaneous rupture of membranes compared to term births in Slovenia, the research delves into the nuanced relationship between circadian disruptions and the timing of childbirth. The investigation encompasses over 2 decades of data, encompassing more than half a million singleton births, aiming to uncover patterns of rhythmicity and their association with varying degrees of prematurity and different types of spontaneous labor onset.

The timing of birth is influenced by two interacting clocks: (1) a developmental clock that measures the overall length of gestation, and (2) a circadian clock that defines when within a 24-h period the birth occurs^14,15^. Animals studied exhibit reliable, species-specific, circadian rhythms at the onset of labor. Interestingly, pregnancy induces an earlier chronotype in mice and in women^16^. After the 24th week of pregnancy in humans, uterine contractile activity shows a diurnal pattern, with 67% of contractions occurring at night. Likewise, clinical data demonstrate that spontaneous rupture of the fetal membranes and onset of labor most commonly occur between late night and early morning (between 11:00 PM and 4:00 AM) in both term and preterm human birth, as reviewed recently^17^. But the results must be viewed also considering confounding factors, such as induced labor and the use of labor analgesia that can affect uterine contractions and, in this way, change the delivery time and the circadian rhythm of childbirth^18^. In a prospective study of over 43,000 deliveries between 2016 and 2018 in USA hospitals, spontaneous vaginal delivery reached the peak at 2:00–4:00 AM which is consistent with some earlier studies in other countries^19^. An even earlier study from the times when there were little or no medical interventions at the labor (and less artificial light) defined the peak of spontaneous labor between midnight and 4:00 AM^20^. It was established that analgesia affects the circadian rhythm of labor and such labor is often aggravated using induction, stimulation, and ending with operative delivery^18^.

The onset of labor is clearly multifactorial where genetic and nongenetic factors play a role. The onset is influenced by both the maternal and fetal hypothalamic–pituitary–adrenal axis (HPA) that regulates hormone-related homeostasis, where cortisol plays a significant role. Not only hormone coordination but also metabolic, endocrine, and circadian systems, are required for a normal pregnancy, including the sleep-promoting hormone melatonin, an essential component to understanding human parturition in a circadian manner^17^.

Despite decades of research into the biological stimuli that trigger human parturition, the exact network and sequence of events are still not fully understood. Even less we understand why some births occur prematurely, i.e., at too early gestational age. Preterm birth (PTB) is defined by World Health Organization as delivery before 37 completed weeks of gestation^21^. Annually, it affects almost 15 million newborns worldwide and is the leading cause of death in children younger than 5 years of age^22^. PTB represents a common and multifactorial condition in which the lifestyle and clinical condition of the mother (like hypertension, diabetes, obesity, etc.), interact with environmental factors, such as shift work, and genetic or epigenetic factors. Recent epidemiological studies have shown that maternal shift work (which causes chrono disruption) is associated with different adverse pregnancy outcomes, including preterm birth^17^.

In this study, we applied the data from the Slovenian National Perinatal Information System (NPIS) of the National Institute for Public Health in Slovenia. The aim of our retrospective study was to investigate rhythmic behavior of different types of preterm births, namely labors initiated with contraction and labors initiated with a spontaneous rupture of membranes, and to investigate rhythmic behavior of spontaneous preterm births, compared to the term births in a 24-h circadian scheme. The study was conducted on the labor onset times obtained from the Slovenian birth cohort data collected from 1990 to 2018, which includes more than 550,000 cases of singleton births. The results are of general importance and represent evidence of gradual disruption of rhythmicity from mild to extreme prematurity. Moreover, we show differences of circadian trends between spontaneous labors initiated with contraction and labors initiated with a spontaneous rupture of membranes. Understanding the circadian behavior of prematurity may have practical value for preventive care in ensuring a successful pregnancy and optimal development of the fetus.

## Methods

### Statistical analysis

We stratified the data in dependence on the labor type in three groups, namely induced labor, spontaneous labor with spontaneous rupture of membranes, and spontaneous labor with contractions.

Preterm birth is defined as babies born alive before 37 weeks of pregnancy. There are sub-categories of preterm birth, based on gestational age. We thus additionally divided the labors regarding gestational age (GA) at delivery in four groups, namely term labor (GA ≥ 37 weeks), preterm labor (including moderate to late preterm labor 32 ≤ GA < 37), very preterm labor (28 ≤ GA < 32) and extremely preterm labor (GA < 28). We calculated the number of the labor onsets for each hour in a day within each of the groups. Furthermore, we analyzed the circadian trends of labor onsets for each group.

We modeled the observed count data (number of labor onsets per hour) with a generalized Poisson distribution linked with the cosinor regression model using a logarithmic link function. The cosinor regression model used in our analysis can be described with the following equation:

$$y(t)={\sum }_{{\text{i}}=1}^{N}\left({A}_{i,1}\cdot \mathit{sin}\left(\frac{t}{24h/i}\cdot 2\uppi \right)+{A}_{i,2}\cdot \mathit{cos}\left(\frac{t}{24h/i}\cdot 2\uppi \right)\right)+ C+e(t),$$

where $N$ is the number of components in the model, *t* presents the time of a day in hours (values from the interval[0, 23]), ${A}_{i,1}$, ${A}_{i,2}$ and $C$ are the regression coefficients, and $e(t)$ is the error term^23,24^. This model can be used to identify rhythmic patterns and to assess the rhythmicity parameters of the observed data. Namely, the cosinor model can be used to evaluate acrophase—a time of a maximal peak in a day, amplitude—a distance between a rhythm-adjusted mean (Midline Statistic Of Rhythm, MESOR) and either extreme, MESOR, and rhythmicity strength—a ratio between the amplitude and MESOR (a graphical representation of the rhythmicity parameters is available as Suppl. Fig. S1).

Cosinor regression presents a robust method that can also be used in scenarios when other methods fail^24^. An additional advantage of the cosinor model is that it can be used to describe rhythmic patterns in which multiple peaks are observed in a single period or when the observed peaks and/or nadirs are asymmetric. This is also the case of the analyzed dataset, where multiple peaks of labor onset counts can be observed in a single day in specific cases (e.g., corresponding with the start of the hospital day and night shift in induced labors). In this case, a multi-component cosinor model can be applied, which describes the observed data with multiple harmonic components.

We started the fitting process of each group of data using a model with a single harmonic component (N = 1). We iteratively added additional components for which the periods were set to 12 h, 8 h, and 6 h, respectively. We compared the simpler models (fewer harmonic components) with more complex models (more harmonic components) using the likelihood ratio test. If the obtained *p* value was lower than 0.05, the more complex model was selected as more suitable. In this manner, we assessed the optimal numbers of harmonic components for each group of data. These numbers were consistent with the main periods in the data observed in Fourier periodograms (see Suppl. Table S1 and Suppl. Figs. S2–S5). The latter was obtained by conducting the Fourier transformation of the data describing the number of labors per hour from the first hour of 1. 1. 1990 (hour 0) to the last hour of 31. 12. 2018 (hour 254,207). Finally, the obtained cosinor models were used to evaluate the rhythmicity as well as the rhythmicity parameters for each group of measurements.

### Institutional review board statement

The data was obtained based on agreement #968-232/2019-1/007 between the National Institute for Public Health an (at that time) doctoral student Urša Kovač, University of Ljubljana, Faculty of Medicine, Slovenia. The subject of the agreement was the transmission of data from the database: Perinatal Information System of the Republic of Slovenia, for the purpose of research: Examination of circadian trends in childbirth in Slovenia by gestational age and onset of labor, in accordance with Article 17 of the Personal Data Protection Act and in accordance with the General Regulation on Personal Data Protection (EUR Lex, 2016/679, GDPR).

## Results

### Data

**Figure 1:**
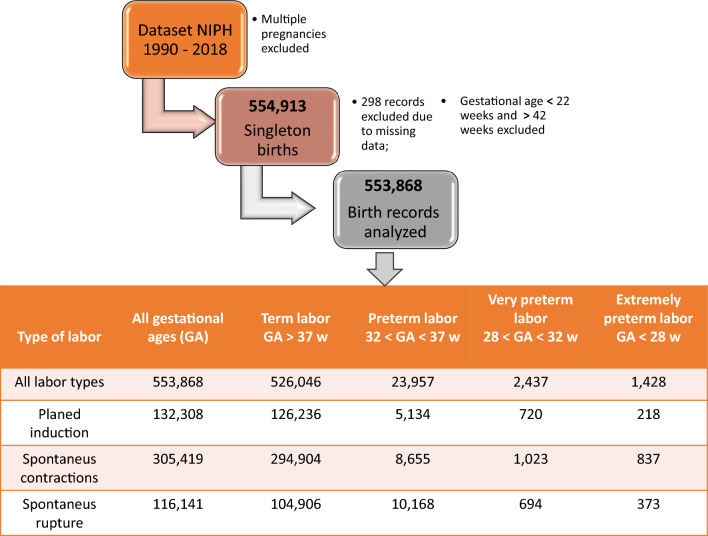
The flow of data with exclusion criteria. The dataset used in the analysis was stratified by gestational age and labor type.

Slovenian birth cohort from 1990 to 2018 was included in this retrospective study. The data were obtained from the Slovenian National Perinatal Information System (NPIS) (National Institute of Public Health (NIJZ)) based on agreement # 968-232/2019-1/007, as detailed in Methods section. The timeline of the sample collection was limited with the data that were available to us through the NPIS at the time when the study was conducted. The study was initiated in 2019, so the latest data available were from 2018 while the registry exists from the year 1990 and on. The inclusion and exclusion criteria of the study were as follows. We included all the data describing singleton births. We excluded the data on stillborn births and the data with missing values of observed variables and anomalies in these variables. There were 554,931 total singleton births in the cohort. 298 records were excluded due to missing relevant data, such as gestational age at delivery, the time of labor, and the time of labor onset. In addition, records with data abnormalities such as gestational age at delivery lower than 22 weeks or higher than 42 weeks were also excluded. In total, 553,868 birth records were analyzed. These data were further stratified by gestational age at delivery and labor type as presented in Fig. 1.

There are several confounders that can affect the time of labor. For example, it has already been reported that women with spontaneous onset of their first labor have longer active labors in comparison to the average labor durations of all delivering women^25,26^. To reduce the effects of such confounding factors, we focused our analysis on the time of labor onset, which presents a more objective measure than the time of labor itself. The stratification of labor types was decided based on our research questions. We wanted to observe the circadian characteristics of different types of spontaneous labors (i.e., labors initiated with contractions and labors initiated with rupture of membranes) in dependence on the gestational age at delivery. To reduce the effects of potential confounders, we observed induced labors separately. These labors could simply be excluded, however, we performed a similar analysis on these as on the spontaneous labors to validate the appropriateness of our methodology, since we knew when the main peak in the number of induced labors should be expected, i.e., in the beginning of the hospital day shift. We additionally stratified different labor types on gestational ages according to their definitions (term, preterm, very preterm, extremely preterm). To not only observe different categories of preterm labor and to more accurately identify the gestational age at which the rhythms are disrupted, we additionally divided the labors based on gestational ages with a 1-week resolution.

### Circadian trends depend on the labor type and on the gestational age at delivery

#### All types of labor

**Figure 2:**
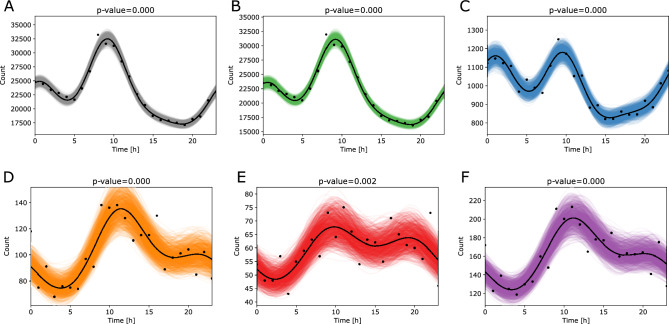
Cosinor regression analysis on the time of labor onset in all the labor types. (**A**) All gestational ages, (**B**) term labors (GA ≥ 37 weeks), (**C**) preterm labors (moderate to late preterm 32 ≤ GA < 37), (**D**) very preterm labors (28 ≤ GA < 32), (**E**) extremely preterm labors (GA < 28), (**F**) very preterm and extremely preterm labors combined (GA < 32). The *p* values correspond to the overall significance of each fit. The time corresponds in hours of the day with 0 h as midnight and 12 h as noon.

We applied the cosinor-based analysis to evaluate the circadian trends of all labor types in dependence on the gestational age at delivery (Table 1 and Fig. 2). This analysis also includes the induced labors where the start of the labor corresponds to the working schedule of the personnel and not to intrinsic endogenous factors that trigger the labor. The times reflect the hour of the day when labor has started (i.e., labor onsets). The number of labors initiated at a given hour of the day was then analyzed for their circadian characteristics. We can see that all labor subtypes have circadian characteristics, based on the *p* values (overall significance of a fit to the cosinor function) and *q* values (adjusted to false discovery rate). An important parameter for comparing the circadian characteristics of labors is the acrophase which describes a time of a day with the maximum number of labor onsets, and the amplitude which shows the size of the effect with the peak in the number of cases. The amplitude is the highest in the morning for all gestational ages, between 9:12 and 11:30. However, the prominence of this peak is smaller for all premature labors, namely, preterm, very preterm, and extremely preterm labors as also seen from the Strength parameter (Table 1). The graphical representation of rhythmicity shows an additional peak around midnight in preterm labors (Fig. 2C). This second peak is shifted towards evening hours in very and extremely preterm labors (Fig. 2D,E).

**Table 1:** Results of the cosinor regression performed on the data that correspond to all the labor types.

GA group	Comp	*p*	*q*	Acrophase[h]	Amplitude	Mesor	Strength	Cases
All	3	0	0	9.2092	7582.091	24,882.77	0.3047	553,868
Term GA ≥ 37	3	0	0	9.2092	7407.325	23,683.73	0.3128	526,046
Preterm 32 ≤ GA < 37	3	0	0	9.5095	176.2316	1003.149	0.1757	23,957
Very preterm 28 ≤ GA < 32	2	0	0	11.5115	30.2865	104.9017	0.2887	2437
Extremely preterm GA < 28	2	0.0024	0.0036	9.8098	9.643	58.1068	0.166	1428
Very or extremely preterm GA < 32	2	0	0	11.2112	38.4105	162.6305	0.2362	3865

*GA* gestational age, *Comp*. the optimal number of components in a cosinor model, *p* the overall significance of a fit, *q* false discovery rate adjustment, *MESOR* midline statistic of rhythm, *Acrophase* time of a maximal peak in a day in hours, *Amplitude* a distance between a rhythm-adjusted mean (*MESOR*) and either extreme, *Strength* a ratio between amplitude and a MESOR and describes a relative strength of rhythmicity, *Cases* number of births in each scenario.

#### Circadian trends of induced labors are not due to intrinsic factors

The analysis of circadian rhythmicity was additionally performed on the data that correspond to the induced labors (Suppl. Fig. S6). We can refer to this as our control analysis since the peaks should be observed at approximately the same time of the day for all gestational ages due to the working schedule of hospital personnel. These expectations were confirmed. The main peak is observed around a narrow time interval, between 9:24 and 10:30. This is in line with the start of the hospital day shift (7–8 AM) when most of the labors are initiated. However, an additional peak occurs in all preterm births (Suppl. Fig. S6C–F), which can be explained by the emergency procedures. In this group we observed the second peak occurring in the evening hours, after 8 PM, likely corresponding to the start of the hospital night shift. In the case of induced labors rhythmicity models for all gestational ages were significant (Suppl. Table S2), however, this must be seen primarily through the eyes of hospital work shifts. To get a better insight into intrinsic factors that trigger the circadian shape of parturition we focused in the continuation on spontaneous labors only.

#### Spontaneous labors

We divided the spontaneous labors into two groups, namely labors initiated with spontaneous contractions and labors initiated with a spontaneous rupture of membranes.

**Figure 3:**
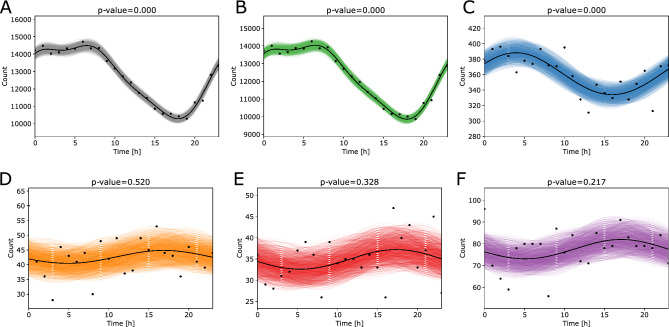
Cosinor regression analysis on the time of labor onset in spontaneous labors that started with contractions. (**A**) All gestational ages, (**B**) term labors (GA ≥ 37 weeks), (**C**) preterm labors (moderate to late preterm 32 ≤ GA < 37), (**D**) very preterm labors (28 ≤ GA < 32), (**E**) extremely preterm labors (GA < 28), F—very preterm and extremely preterm labors combined (GA < 32). The *p* values correspond to the overall significance of each fit. The time corresponds in hours of the day with 0 h as midnight and 12 h as noon.

We first describe the analysis of the labors initiated with spontaneous contractions (Fig. 3). Our results indicate that the circadian trends are disrupted in very and extremely preterm groups (Fig. 3D,E). Due to the low number of labors in very and extremely preterm groups, we additionally analyzed these two preterm groups together (Fig. 3F). However, the circadian trends were absent also in this case. Further details describing the regression results are available in Table 2.

**Table 2:** Results of the cosinor regression performed on the data that correspond to the labors initiated with contractions.

GA group	Comp	*p*	*q*	Acrophase[h]	Amplitude	Mesor	Strength	Cases
All	3	0	0	6.5065	2099.069	12,392.87	0.1694	305,419
Term GA ≥ 37	3	0	0	6.4064	2084.75	11,953.21	0.1744	294,904
Preterm 32 ≤ GA < 37	1	0	0.0001	4.004	27.0332	361.1317	0.0749	8655
Very preterm 28 ≤ GA < 32	1	0.5203	0.6244	16.9169	2.1949	42.6533	0.0515	1023
Extremely preterm GA < 28	1	0.3278	0.4185	17.3173	2.3115	34.9133	0.0662	837
Very or extremely preterm GA < 32	1	0.2171	0.2895	17.1171	4.4355	77.5635	0.0572	1860

*GA* gestational age, *Comp*. the optimal number of components in a cosinor model, *p* the overall significance of a fit, *q* false discovery rate adjustment, *MESOR* midline statistic of rhythm, *Acrophase* time of a maximal peak in a day in hours, *Amplitude* a distance between a rhythm-adjusted mean (*MESOR*) and either extreme, *Strength* a ratio between amplitude and a MESOR and describes a relative strength of rhythmicity, *Cases* number of births in each scenario.

Additionally, we separated the labors initiated with spontaneous contractions by weeks presenting the gestational age. We analyzed the circadian trends in the interval from 41 to 34 weeks. The analysis revealed that circadian trends and rhythmicity are disrupted in contractions-initiated spontaneous labors between week 35 and week 36 (Suppl. Fig. S7). For the gestational ages equal to or above the 36th week, circadian models were significant, and the main peak occurred early in the morning (between 6 and 7 AM). However, this peak was less prominent than in the labors initiated with a spontaneous rupture of membranes (Suppl. Fig. S8).

**Figure 4:**
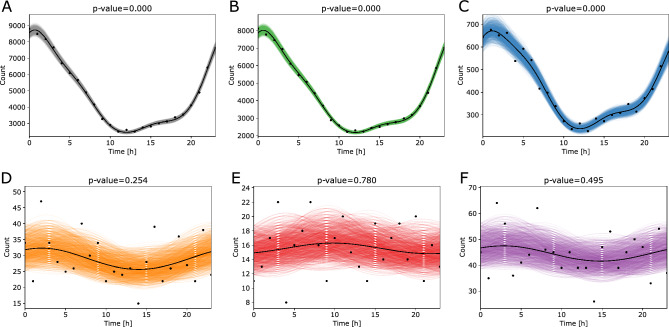
Cosinor regression analysis on the time of labor onset in spontaneous labors that started with spontaneous rupture of membranes. (**A**) All gestational ages, (**B**) term labors (GA ≥ 37 weeks), (**C**) preterm labors (moderate to late preterm 32 ≤ GA < 37), (**D**) very preterm labors (28 ≤ GA < 32), (**E**) extremely preterm labors (GA < 28), (**F**) very preterm and extremely preterm labors combined (GA < 32). The *p* values correspond to the overall significance of each fit. The time corresponds in hours of the day with 0 h as midnight and 12 h as noon.

In the case of spontaneous labors that are initiated with a spontaneous rupture of membranes (Fig. 4), the circadian trends were again disrupted in very preterm labors (Fig. 4D), but were observed at earlier gestational ages than in spontaneous labors initiated with contractions, namely, from the week 32 onwards (Suppl. Fig. S8). When observing the circadian trends in refined gestational age groups, whereas each week was considered as a separate group, we extended the observed intervals to include the gestational ages as well from the 31^st^ week onwards, where the rhythmicity was disrupted. It is interesting to note that the main peak of the number of cases was detected much earlier than in the spontaneous labors initiated with contractions. While the main peak in the case of spontaneous labors that are initiated with a spontaneous rupture of membranes was detected around 1 AM (Fig. 4), the main peaks in the case of spontaneous labors initiated with contractions occur between 6 and 7 AM (Fig. 3). However, even in the former case, the peak becomes less prominent in very and extremely preterm groups. Further details describing the results of the regression are available in Table 3.

**Table 3:** Results of the cosinor regression performed on the data that correspond to the labors initiated with a rupture of a membrane.

GA group	Comp	*p*	*q*	Acrophase[h]	Amplitude	Mesor	Strength	Cases
All	3	0	0	0.7007	3130.908	5594.679	0.5596	116,141
Term GA ≥ 37	3	0	0	0.7007	2912.679	5100.049	0.5711	104,906
Preterm 32 ≤ GA < 37	3	0	0	1.2012	215.1455	455.0877	0.4728	10,168
Very preterm 28 ≤ GA < 32	1	0.2537	0.3295	2.002	3.3261	29.0123	0.1146	694
Extremely preterm GA < 28	1	0.7799	0.7999	9.8098	0.7432	15.5506	0.0478	373
Very or extremely preterm GA < 32	1	0.4949	0.6044	2.8028	2.9548	44.5074	0.0664	1067

*GA* gestational age, *Comp*. the optimal number of components in a cosinor model, *p* the overall significance of a fit, *q* false discovery rate adjustment, *MESOR* midline statistic of rhythm, *Acrophase* time of a maximal peak in a day in hours, *Amplitude* a distance between a rhythm-adjusted mean (*MESOR*) and either extreme, *Strength* a ratio between amplitude and a MESOR and describes a relative strength of rhythmicity, *Cases* number of births in each scenario.

#### Circadian trends depend on the labor type and on the gestational age at delivery

**Figure 5:**
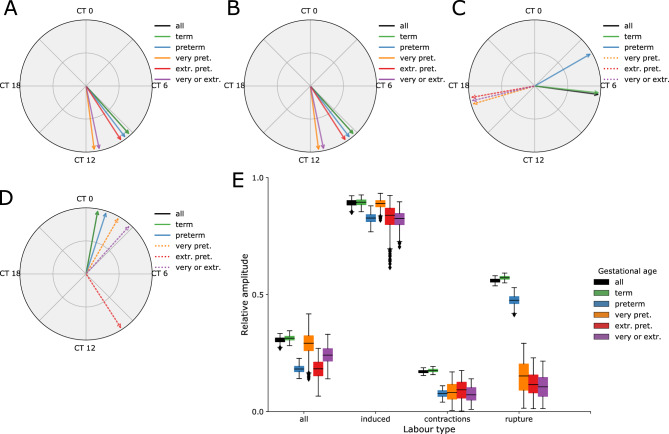
Acrophase plots in dependence on the labor types and gestational ages at the time of delivery (**A**–**D**) and relative rhythmicity amplitudes (**E**). (**A**) All labor types, (**B**) induced labors, (**C**) labors initiated with contractions, (**D**) labors initiated with a spontaneous rupture of membranes, (**F**) relative rhythmicity amplitudes. Relative rhythmicity amplitudes present the percentage of count data that can be explained with the rhythmic behavior. The results of significant rhythmicity models (FDR adjusted *p* value < 0.05) are presented with solid lines and the results of nonsignificant models with dashed lines. Different colors indicate the type of birth based on gestational age: black—all birth types; green—term (GA ≥ 37 weeks); blue—preterm (moderate to late preterm 32 ≤ GA < 37); orange—very preterm (28 ≤ GA < 32); red—extremely preterm (GA < 28); purple – very or extremely preterm births combined (GA < 32); CT: circadian time.

The acrophase is defined as the time point in a cycle during which the cycle peaks. The acrophase plots can be used to analyze the similarities and differences between different gestational ages and labor types (Fig. 5). Similar trends are observed in the case when all labor types are combined (Fig. 5A) and in the case of induced labors (Fig. 5B). Moreover, differences in the context of peak occurrence between different gestational ages are relatively small in these two scenarios. In both groups, peaks always occur between 9 AM and 12 PM, and the rhythmicity is always significant.

Larger differences are observed between the labors initiated with contractions (Fig. 5C) and labors initiated with spontaneous rupture of membranes (Fig. 5D). In the former, peaks occur between 6 and 7 AM for term labors and around 4 AM for preterm labors. Circadian rhythmicity is disrupted in very preterm and extremely preterm labors, which means that the time of the peak is not significant either. In the case of labors initiated with spontaneous rupture of membranes (Fig. 5D) significant peaks always occur between 0 and 2 AM. Smaller gestational ages cause the right shifts of the peak, i.e. peaks are delayed towards the dawn. Circadian rhythmicity is again disrupted in very and extremely preterm labors (Tables 2 and 3).

We additionally analyzed the prominence of oscillation amplitudes (Fig. 5E). We divided the evaluated amplitude with an evaluated MESOR (Midline Estimating Statistic of Rhythm) for each of the analyzed combinations to obtain the relative (normalized) rhythmicity amplitudes. Relative rhythmicity amplitudes define the percentage of the count data that can be explained with the rhythmic behavior. High relative amplitude values indicate strong rhythmicity while low amplitudes indicate weak or absent rhythm. The assessed number of harmonic components in a model was consistent with the periods in the data that were observed in the Fourier periodograms (Suppl. Figs. S2–S5). Relative rhythmicity amplitudes for the analyzed scenarios are presented in Fig. 5E.

## Discussion

Herein we present the first study on a large retrospective dataset of over half million singleton births in Slovenia focusing on the circadian rhythmicity and the ways in which this varies by the onset of labor and by the gestational age at delivery. In the first set of analyses, we observed the circadian patterns of labor onsets in different gestational age groups independent of the type of labor. Next, we observed onsets of induced labors separately from spontaneous labors to reduce the effects of potential confounders. Induced labors could simply be excluded. However, we performed their thorough analysis as well to validate the appropriateness of our methodology, since we knew when the peaks in the number of induced labors are to be expected, i.e., in the beginning of the day and in the beginning of the night shift. We additionally observed different types of spontaneous labors (i.e., labors initiated with spontaneous contractions and labors initiated with spontaneous rupture of membranes), since these two labor types are initiated by different mechanisms and might reflect different patterns as was also demonstrated by the obtained results. It has previously been reported that the duration of labor significantly differs between nulliparas and multiparas^25,26^. To diminish the influence of specific confounding factors, such as the number of children, a woman has already given birth to, we focused on labor onset instead of the time of the labor. For the spontaneous labors, the labor onset was observed as the time of the rupture of membrane, if labor was started with the rupture, or the time when contractions started if the labor was started with the contractions.

When all labor types are combined, rhythmicity models of all gestational age groups are significant. We observed the peaks in the number of cases around 10 AM for all gestational ages. The prominence of this peak is smaller for the premature labors, namely, preterm, very preterm, and extremely preterm labors (Fig. 2C,D). An additional peak ranging from 8 PM to 1 AM is also observed, whereas its exact location is dependent on the gestational age at the delivery. This set of data also included the induced labors, where the diurnal behavior is most likely due to the working schedule of hospital personnel and not of the intrinsic circadian nature. Consequently, the induced labors group was considered as a control group to spontaneous labors with contractions or spontaneous rupture of membranes.

When observing spontaneous labors only, the circadian disruption occurs with very preterm gestational ages irrespective of the type of spontaneous labor (Figs. 3 and 4). The labors initiated with spontaneous contractions showed the disturbance or absence of circadian trends in very and extremely preterm groups (Fig. 3). Since also analyzed both groups together (very and extremely preterm labors combined), and additionally separated the data per each week of prematurity. Analysis revealed that the circadian trends are disrupted at week 35 where the *p* value for circadian behavior lost the statistical significance (*p* = 0.066). From 38th weeks onward the main peak of labors occurred between 6 and 7 AM while the nadir was between 5 PM and 7 PM (Suppl. Fig. S7).

Labors with spontaneous rupture of membranes have a distinct circadian behavior that is time advanced compared to the pattern observed with spontaneous contractions. Most labors with rupture onset around 1 AM. The circadian trends were again disrupted in very preterm labors (Fig. 4), but were observed at earlier gestational ages than in spontaneous labors initiated with contractions. While at 32 weeks the circadian behavior is still observed (*p* = 0.0), it is lost in labors at 31 weeks (*p* = 0.760) (Suppl. Fig. S8).

With the acrophase plots, we identified a more precise timing that distinguishes between the peaks of the term (between 6 and 7 AM) and preterm (4 AM) spontaneous labors initiated with contractions (Fig. 5C) while in labors initiated with spontaneous rupture of membranes significant peaks occur between 0 and 2 AM and prematurity results in peak delay towards the dawn (Fig. 5D). It is interesting to observe that prematurity results in advancing the peaks of labors starting with contractions and delaying the peaks of labors starting with spontaneous rupture of membranes.

The absence of circadian rhythms is observed in extremely preterm deliveries. This could be explained with fetal hypothalamic–pituitary–adrenal axes immaturity, which normally mediates the diurnal variation in uterine contractions and pathological rather than biological mechanisms such as infections leading towards premature spontaneous rupture of membranes^1^.

Our observations regarding the disruption of circadian trends in very preterm labors are similar to the results of the study conducted by Luque-Fernandez et al. on the dataset describing 395 singelton births in Lima, Peru, between 2009 and 2010^1^. The authors report on disrupted circadian rhythmicity in cases of extremely preterm spontaneous rupture of membranes (78 cases). In the case of premature spontaneous rupture of membranes, they observed peaks between 5 and 10 AM for preterm labors between 32 and 37 weeks while in our case the analysis revealed the main peak around 1 AM (Fig. 4C). These differences could be partially explained by a smaller sample size of the study by Luque-Fernandez et al. that included only 154 cases while our analyses were performed on 10,168 preterm labors with spontaneous rupture of membranes. Small sample sizes might lead to misleading results, as a relatively small number of events can alter the conclusions drawn from the data. Furthermore, Luque-Fernandez et al. additionally performed a replication analysis on the data from the National Collaborative Perinatal Project (NCPP) describing a prospective pregnancy cohort study conducted in United States between 1959 and 1966^27^. In the latter case the observed peaks of premature spontaneous rupture of membranes shifted towards early morning hours, i.e., approximately to 3 AM, for preterm labors between 32 and 37 weeks (1736 cases). Even though these results are closer to the results obtained on the Slovenian cohort, additional analyses performed on different cohorts from different regions should be conducted to provide a thorough explanation for different patterns of circadian response observed in different geographical locations and different populations.

The study conducted by Martin et al. found that the number of preterm births after spontaneous onset does not vary by time of day nor do they reduce during weekend or holidays^28^. However, in their study, the timing of birth (and not the onset of labor) was analyzed, which can decrease the significance of the obtained results due to additional confounders affecting the labor duration, like analgesia or stimulation of labor. Moreover, they did not separate among different types of spontaneous labors, for which different trends might be exhibited as demonstrated by our results.

The burden of preterm birth is considerable and is still growing despite the efforts of obstetricians to prevent it. Improved recording of all pregnancy outcomes and standard application of preterm definitions is important. Distinguishing between spontaneous preterm labors as well as among spontaneous preterm labors and induced labors is important for the successful monitoring of trends. These trends can be further used to identify and study the potential causes of preterm birth.

We did not study the role of oxytocin and melatonin in the circadian regulation of birth onset, even though we know they play an important role as emphasized by others^28^. The parallel upregulation of myometrial receptors for melatonin and oxytocin at the time of labor onset underscores the importance of the melatonin signal for uterine contractions in the human parturition^29,30^. Interestingly, the recurring nocturnal signal provided by circulating melatonin to the uterus is ineffective for preterm pregnancy. This is clearly not due to the absence of the hormone since human melatonin secretory rhythms continue throughout pregnancy and indeed melatonin levels keep high amplitude during late pregnancy^31,32^. More likely, the myometrial expression of melatonin receptors during most of the pregnancy appears to be low, much like the oxytocin receptors. However, the expression of these receptors prematurely in the myometrium of pregnant women might contribute to preterm labor in at least some cases. Given the growing evidence for a genetic predisposition to preterm birth in some families, it may prove valuable to explore potential genetic associations between preterm labor and melatonin receptor polymorphisms^33^.

Interestingly, when observing all types of labor combined, we see two peaks in the number of cases during the day, one in the late morning, around 10 AM, and the other at midnight. This observation could be explained by the rising number of obstetric interventions (elective cesarean sections, labor inductions, etc.). The predominant nocturnal pattern seems to have disappeared in a Spanish highly medicalized population^34^. A study conducted in the Netherlands suggested that the time of birth is also affected by the care provider, showing that births of women cared for by midwives peaked earlier in the day compared to women cared for by obstetricians^35^. Timing of birth can also be dependent on weather, season of the year, and lunar phases^36^. However, the hallmark of primate nocturnal deliveries is evident when multiple births, malpresentations, cesarean sections, and vaginal interventions are excluded. High rates of different obstetrics interventions might alter circadian patterns of labor onset. Prematurity is caused by various pathophysiological processes, but it is also important to recognize the importance of circadian rhythms and their loss in very preterm labors. The contribution of this article is certainly an important building block for the understanding of such an important problem as premature birth.

## Conclusion

We presented the first study based on the dataset obtained from the National perinatal information system in Slovenia and focusing on circadian rhythmicity and the ways in which this varies by the type of the onset of labor and by the gestational age at delivery. The advantage of the study is the application of a large dataset of more than 500,000 cases of singleton births during a 28-year period that offers a large enough number of births to obtain relevant results that are likely not limited to the Slovenian population and are of a more general importance. Another advantage is that we were able to separate between different types of spontaneous labors, which has not been performed in other large-scale cohort studies on circadian behavior in the past^28^. The timing of spontaneous labors varies by different components, one of them is the onset of labor. The onset of labor is multifactorial, with genetic and nongenetic factors playing important roles. It has great implications and can be used as well to identify pathological mechanisms in preterm birth^1^. According to our knowledge, this is the first study demonstrating different circadian trends in different types of spontaneous labors, while additionally investigating the circadian trends of labor onsets in dependence on gestational age at delivery. From the limitation standpoint, our study would benefit greatly if it could be compared directly to other cohorts from European and non-European populations, to validate the generality of our conclusions. Unfortunately, this is not possible at present and remains to be addressed in the future. However, we believe that due to the size of the cohort and the retrospective data covering over 20 years our analyses do offer novel insights into circadian patterns of preterm births depending on the type of onset that are of general importance. Among the open questions for further research and analyses is, for example, how circadian rhythmicity varies between different maternity units and social and/or demographic groups? And finally, what are the biological mechanisms behind the triggers of spontaneous labors? It would also be important to understand why labors starting with spontaneous rupture of membranes peak earlier (1 AM) than labors starting with contractions (between 6 and 7 AM) and why they reflect circadian trends at earlier gestational ages of prematurity.

Even though specific measures were taken to reduce the effects of potential confounders, certain confounders could still influence the obtained results. For example, a disrupted circadian pattern of labor onset could as well be observed due to other factors, such as disrupted circadian rhythm of a mother, due to other circumstances, such as night shift work of the mother. Nevertheless, we still believe that our study provides robust results, due to a relatively small fraction of such potential cases. Another possible confounder that could affect the obtained results is the age of the mother at the delivery. For labors initiated with spontaneous contractions, the average age of a mother in an extremely preterm group equals 28.72 years and is only to small degree higher than the very preterm, preterm and term group (28.21 years, 28.1 years, and 28.03 years, respectively). However, differences are slightly larger for labors initiated with spontaneous rupture of membrane. In this case, the average age of a mother in the extremely preterm group equals 30.07 years, 29.4 years in the very preterm group, 29.07 years in the preterm group and 28.55 years in the term group. The age of the mother at childbirth could thus play a role in the gestational age at delivery and in the circadian rhythm of the onset of the labor and should be investigated further in future studies. Namely, a potential study could observe not only how does gestational age affects the circadian rhythm of the onset of delivery but also how do factors, such as age of the mother at the birth, affect trends in gestational ages at delivery.

Incorporating knowledge of circadian rhythm disruption in preterm labor into hospital practice and prenatal care strategies requires a multidisciplinary approach involving obstetricians, nurses, sleep specialists, nutritionists, and researchers. By integrating knowledge of circadian disruptions into clinical practice, healthcare providers may be able to offer pregnant women more personalized and effective care, ultimately contributing to better maternal and fetal outcomes.

The hospital environment can be optimized to support natural circadian rhythms. This could mean controlling exposure to artificial light at night, particularly in maternity wards, and encouraging exposure to natural light during the day to help regulate patients' internal clocks. Hospitals could adapt the timing of administration of drugs to manage preterm labor to the patient's individual circadian rhythm. Clinicians could incorporate circadian profiling into prenatal assessments. Knowing a person's circadian rhythm could help tailor treatment plans specifically to their internal clock, potentially optimizing the effectiveness of treatments aimed at preventing preterm labor. Hospital monitoring systems could be designed to consider circadian variations in physiological parameters and time monitoring interventions to coincide with times when certain physiological changes associated with labor are more likely to occur due to circadian rhythms. Prenatal care programs could also include education on the importance of regular sleep habits, good sleep hygiene during pregnancy, and establishing a consistent bedtime routine to promote healthy circadian rhythms. On the other hand, advice on meal timing and nutrition could be designed to support circadian rhythms by recommending meal timing to promote hormonal regulation and optimal energy levels throughout the day. Further research into circadian rhythms and their impact on pregnancy could lead to the development of specific interventions and technologies. These could range from wearable devices that monitor and analyze a person's circadian patterns to precision medicine approaches that target specific circadian-related mechanisms to prevent preterm labor.

### Supplementary Information

Supplementary Information.

## Data Availability

The data used in this study are available upon request to the National Institute for Public Health (Slovenia). The recipient may publish or in any other way disclose data to any third party only in the form of results that statistically treat the data in accordance with the National Institute for Public Health (Slovenia), provisions of ZVOP-2 and GDPR.
