# Supplementary material for: Circadian characteristics of term and preterm labors
Source: Sci Rep. 2024 Feb 18;14:4033. doi: 10.1038/s41598-024-54490-2 (PMC10874972; doi:10.1038/s41598-024-54490-2)

**Supplementary materials: Circadian trends in term and preterm labors**

Miha Moškon ^1^, Urša Kovač ^2^, Lucija Raspor Dall’Olio^2^, Ksenija Geršak ^3^, Gorazd Kavšek ^3^, Eva Bojc ^3^, Andreja Trojner Bregar ^3, *^ and Damjana Rozman ^2,^*

^1^ Faculty of Computer and Information Science, University of Ljubljana, Ljubljana, Slovenia

^2^ Centre for Functional Genomics and Bio-Chips, Institute for Biochemistry and Molecular Genetics, Faculty of Medicine, University of Ljubljana, Ljubljana, Slovenia

^3^ University Medical Centre Ljubljana, Division of Gynaecology and Obstetrics, Research Unit, Ljubljana, Slovenia

^4^ University of Ljubljana, Faculty of Medicine, Department of Gynaecology and Obstetrics, Ljubljana, Slovenia

***** Correspondence: [damjana.rozman@mf.uni-lj.si](mailto:damjana.rozman@mf.uni-lj.si) (DR); [andreja.trojner@kclj.si](mailto:andreja.trojner@kclj.si) (ATB)

*Corresponding authors

# **Computational methods**

The fitting was performed using a multi-component cosinor model, that can be described with the following equation:

$$Y=\sum_{i=1}^{N} \left( A_{i,1}\cdot\sin\left( \frac{X}{24/i}\cdot2\pi\right)+A_{i,2}\cdot\cos\left( \frac{X}{24/i}\cdot2\pi\right) \right)+ C,$$

where $N$ is the number of components and X presents a vector of numbers of observed cases at each hour of a day. For each of the scenarios, we started the fitting process with a single-component model *(*$N=1$) and added an additional component in each iteration ($N=N +1$). In each iteration we compared the simpler model with a more complex one using the likelihood ratio test. **More complex model was selected as a more appropriate when the obtained p-value was lower than 0.05. If the more complex model was rejected the fitting process was stopped for the selected scenario and the simpler model was selected as the most suitable.**

**The selected model was used to further analyse the rhythmicity of the observed data as well as their rhythmicity parameters**, namely acrophase – a time of a maximal peak in a day, amplitude – a distance between a rhythm adjusted mean (Midline Statistic Of Rhythm*,* MESOR) and to either extreme, MESOR, and rhythmicity strength – a ratio between an amplitude and a MESOR.

# **Supplementary Tables and Figures**

**Table S1: Five most prominent periods identified using the Fourier periodogram.**

| **Labor types** | **Gestational age** | **Periods** |
| --- | --- | --- |
| All | All | 24.0, 12.0, 8.0, 27.999559422843923, 21.000247831474596 |
|  | Term  GA ≥ 37 | 24.0, 12.0, 8.0, 27.999559422843923, 33.598731165741476 |
|  | Preterm  32 ≤ GA < 37 | 24.0, 12.0, 8.0, 27.999559422843923, 11.200070493897872 |
|  | Very preterm  28 ≤ GA < 32 | 24.0, 6.721878470569569, 3.3148773586135847, 12.0, 3.2316904183775947 |
|  | Extremely preterm  GA < 28 | 3.2565718677940043, 5.225456339417859, 2.4545507212791846, 2.238929011802008, 10.475460501916183 |
|  | Very or extremely preterm  GA < 32 | 24.0, 8.964875158696572, 3.045136559655007, 4.960736866755132, 4.297320598427859 |
| Induced labor | All | 24.0, 12.0, 8.0, 27.999559422843923, 21.000247831474596 |
|  | Term  GA ≥ 37 | 24.0, 12.0, 8.0, 27.999559422843923, 21.000247831474596 |
|  | Preterm  32 ≤ GA < 37 | 24.0, 12.0, 8.0, 23.99773435287454, 27.999559422843923 |
|  | Very preterm  28 ≤ GA < 32 | 24.0, 12.0, 23.99773435287454, 2.6583843137254903, 4.34513879392862 |
|  | Extremely preterm  GA < 28 | 24.0, 8.642121366649668, 12.0, 8.034386852085968, 25.71133812076464 |
|  | Very or extremely preterm  GA < 32 | 24.0, 12.0, 23.99773435287454, 2.6583843137254903, 2.3343036335754492 |
| Contractions | All | 24.0, 24.002266074969317, 12.0, 8.0, 27.999559422843923 |
|  | Term  GA ≥ 37 | 24.0, 24.002266074969317, 12.0, 8.0, 21.000247831474596 |
|  | Preterm  32 ≤ GA < 37 | 2.040634808505856, 24.0, 2.414681408868118, 4.895299351036993, 2.3605972810340985 |
|  | Very preterm  28 ≤ GA < 32 | 5.513555719429141, 6.758694033818995, 2.8598042524468443, 2.0813689769517336, 2.4279656160458454 |
|  | Extremely preterm  GA < 28 | 3.3184690094512033, 5.225456339417859, 5.823646651852191, 2.3270383830246884, 10.424769325404961 |
|  | Very or extremely preterm  GA < 32 | 3.3184690094512033, 4.185596206408272, 5.354565560821484, 7.1704840347512135, 2.293488753958444 |
| Spontaneous rupture of membranes | All | 24.0, 12.0, 8.0, 23.99773435287454, 24.002266074969317 |
|  | Term  GA ≥ 37 | 24.0, 12.0, 8.0, 23.99773435287454, 24.002266074969317 |
|  | Preterm  32 ≤ GA < 37 | 24.0, 12.0, 23.99773435287454, 24.002266074969317, 3.644297899792129 |
|  | Very preterm  28 ≤ GA < 32 | 4.0696721311475414, 2.1474260420010474, 2.456851810687259, 3.078920594932416, 2.5576561258061594 |
|  | Extremely preterm  GA < 28 | 2.741614719268351, 5.1561397103565785, 3.0237659093612463, 2.0402581142251757, 5.864218321068537 |
|  | Very or extremely preterm  GA < 32 | 2.2131216046803175, 3.5835236403620065, 2.785749509605164, 2.762800099988045, 7.930617083671304 |
| Contractions or spontaneous rupture of membranes | All | 24.0, 12.0, 8.0, 24.002266074969317, 6.0 |
|  | Term  GA ≥ 37 | 24.0, 12.0, 8.0, 24.002266074969317, 6.0 |
|  | Preterm  32 ≤ GA < 37 | 24.0, 12.0, 2.972150122763942, 2.109469910711322, 2.3035058945060123 |
|  | Very preterm  28 ≤ GA < 32 | 3.5508869953904174, 2.2131216046803175, 3.2620462215606514, 6.721878470569569, 2.0908358145120167 |
|  | Extremely preterm  GA < 28 | 3.2565718677940043, 5.225456339417859, 3.0024448722642827, 2.238929011802008, 5.92877300184248 |
|  | Very or extremely preterm  GA < 32 | 4.932533907677979, 3.0024448722642827, 3.3184690094512033, 2.7913473152520036, 2.238929011802008 |

**Table S2:** **Results of the cosinor regression performed on the data that correspond to the artificially induced labors.** GA: gestational age*; Comp*. - optimal number of components in a cosinor model; *p* - overall significance of a fit; *q* - false discovery rate adjustment; MESOR: Midline Statistic Of Rhythm; Acrophase - time of a maximal peak in a day in hours; *Amplitude* - a distance between a rhythm adjusted mean (*MESOR*) and to either extreme; *Strength* - a ratio between an amplitude and a MESOR and describes a relative strength of rhythmicity; Cases - number of births in each scenario.

| **GA group** | **Comp.** | **p** | **q** | **Acrophase** | **Acrophase [h]** | **Amplitude** | **MESOR** | **Strength** | **Cases** |
| --- | --- | --- | --- | --- | --- | --- | --- | --- | --- |
| all | 3 | 0 | 0 | -2.4896 | 9.5095 | 7816.926 | 8771.711 | 0.8912 | 132308 |
| term | 3 | 0 | 0 | -2.4634 | 9.4094 | 7541.831 | 8433.456 | 0.8943 | 126236 |
| preterm | 3 | 0 | 0 | -2.673 | 10.2102 | 250.9519 | 304.0449 | 0.8254 | 5134 |
| very preterm | 3 | 0 | 0 | -2.8041 | 10.7107 | 34.1755 | 38.4818 | 0.8881 | 720 |
| extremely preterm | 3 | 0 | 0 | -2.5682 | 9.8098 | 8.7833 | 10.6207 | 0.827 | 218 |
| very or extremely preterm | 3 | 0 | 0 | -2.7516 | 10.5105 | 42.3545 | 48.6143 | 0.8712 | 938 |

# **Figure S1.** Graphical representation of circadian rhythm modelled by cosinor analysis and definition of rhythm features. The **MESOR** is a mean value based on the distribution of values over the cycles of the circadian rhythm, computed using a cosine function. The **amplitude** (A) of the rhythm represents the difference between the peak value and the MESOR value. The **acrophase** is a time period during which the cycle peaks to the upper part of a cosine (or sine) wave fitted to a measurement of a circadian rhythm. The circadian **period** (t) a corresponds to the time distance between the two peaks of a cosine (or sine) wave fitted to a measurement of a circadian pattern.

Figure S2: **Fourier periodograms obtained on the data that correspond to all the labor types.** Figure A presents the periodogram obtained from all gestational ages, Figure B from term labors (GA ≥ 37 weeks), Figure C from preterm labors (moderate to late preterm 32 ≤ GA < 37), Figure D from very preterm labors (28 ≤ GA < 32), Figure E from extremely preterm labors (GA < 28), and Figure F from very preterm and extremely preterm labors combined (GA < 32). Horizontal dashed lines present the significance thresholds (p = 0.05). PSD: power spectral density.

| A | B |
| --- | --- |
| 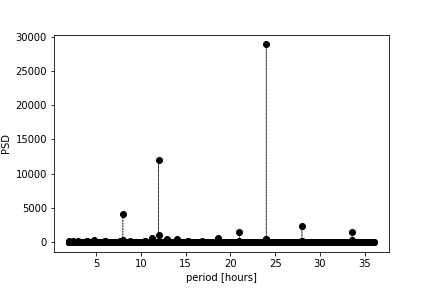 | 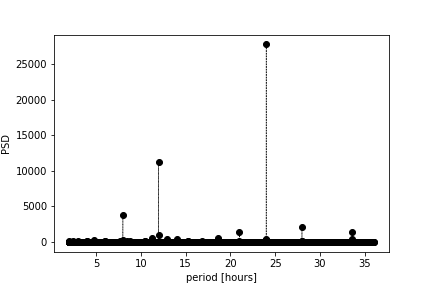 |
| C | D |
| 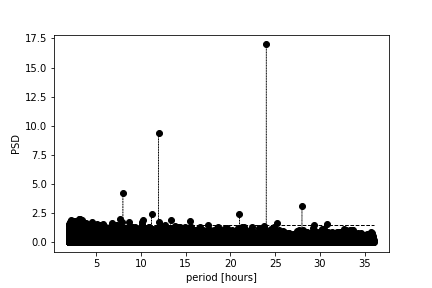 | 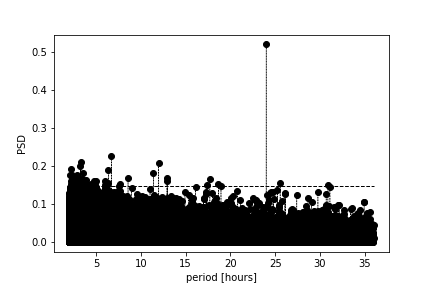 |
| E | F |
| 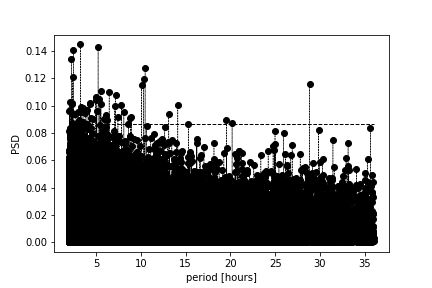 | 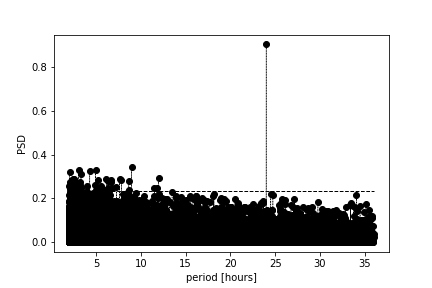 |

Figure S3: **Fourier periodograms obtained on the data that correspond to the artificially induced labors.** Figure A presents the periodogram obtained from all gestational ages, Figure B from term labors (GA ≥ 37 weeks), Figure C from preterm labors (moderate to late preterm 32 ≤ GA < 37), Figure D from very preterm labors (28 ≤ GA < 32), Figure E from extremely preterm labors (GA < 28), and Figure F from very preterm and extremely preterm labors combined (GA < 32). Horizontal dashed lines present the significance thresholds (p = 0.05). PSD: power spectral density.

| A | B |
| --- | --- |
| 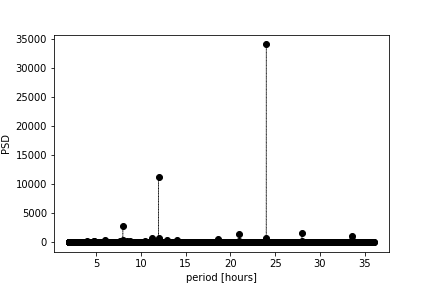 | 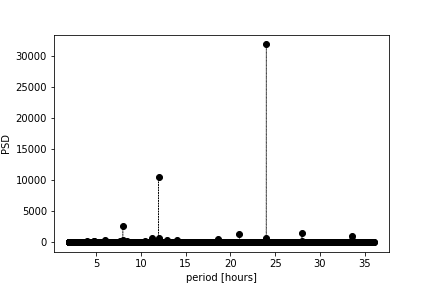 |
| C | D |
| 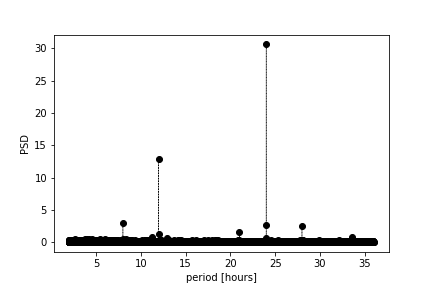 | 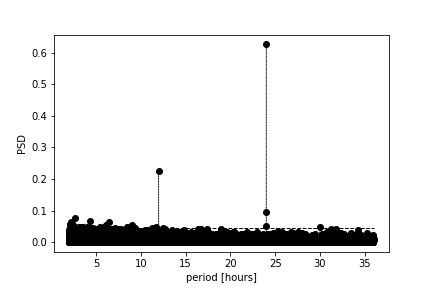 |
| E | F |
| 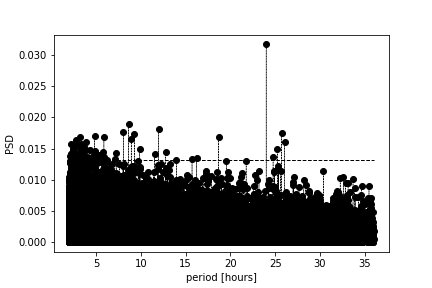 | 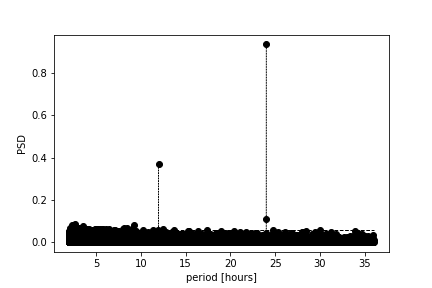 |

Figure S4: **Fourier periodograms obtained on the data that correspond to the labors initiated with contractions.** Figure A presents the periodogram obtained from all gestational ages, Figure B from term labors (GA ≥ 37 weeks), Figure C from preterm labors (moderate to late preterm 32 ≤ GA < 37), Figure D from very preterm labors (28 ≤ GA < 32), Figure E from extremely preterm labors (GA < 28), and Figure F from very preterm and extremely preterm labors combined (GA < 32). Horizontal dashed lines present the significance thresholds (p = 0.05). PSD: power spectral density.

| A | B |
| --- | --- |
| 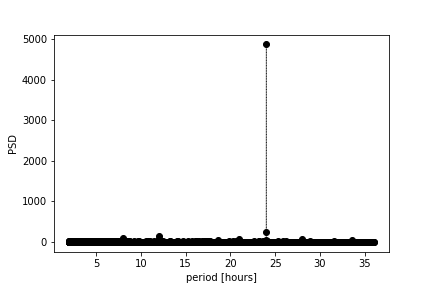 | 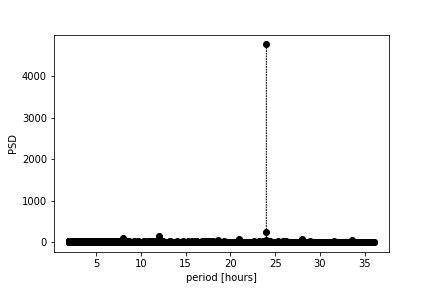 |
| C | D |
| 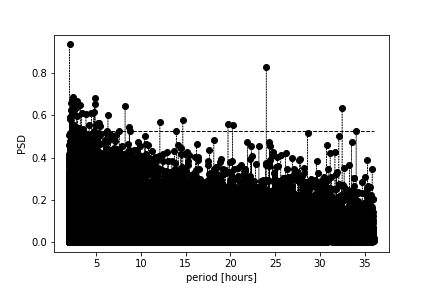 | 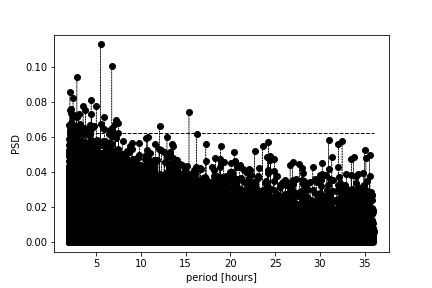 |
| E | F |
| 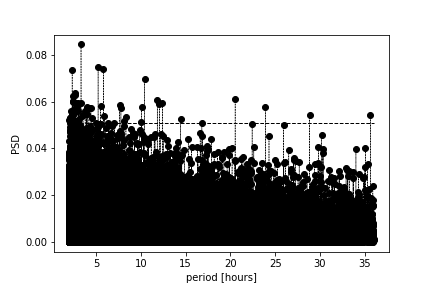 | 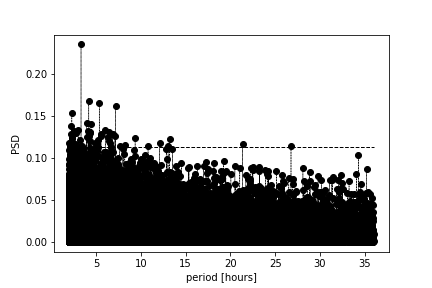 |

Figure S5: **Fourier periodograms obtained on the data that correspond to the labors initiated with spontaneous rupture of membranes.** Figure A presents the periodogram obtained from all gestational ages, Figure B from term labors (GA ≥ 37 weeks), Figure C from preterm labors (moderate to late preterm 32 ≤ GA < 37), Figure D from very preterm labors (28 ≤ GA < 32), Figure E from extremely preterm labors (GA < 28), and Figure F from very preterm and extremely preterm labors combined (GA < 32). Horizontal dashed lines present the significance thresholds (p = 0.05). PSD: power spectral density.

| A | B |
| --- | --- |
| 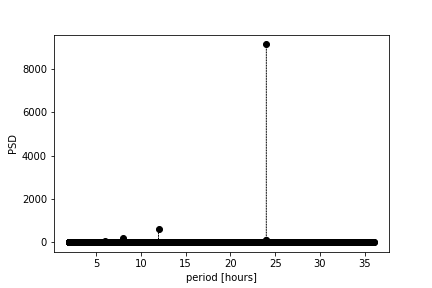 | 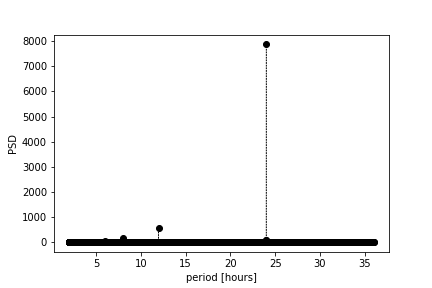 |
| C | D |
| 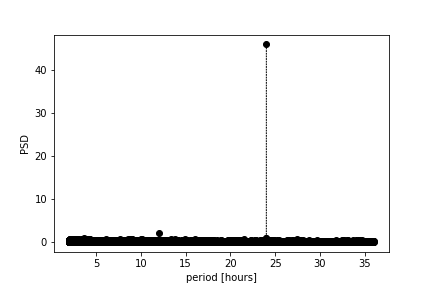 | 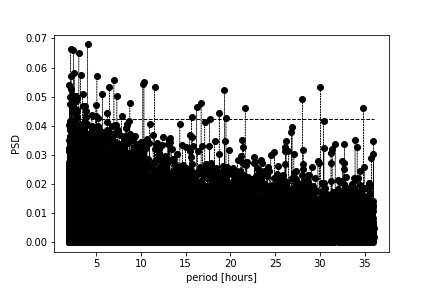 |
| E | F |
| 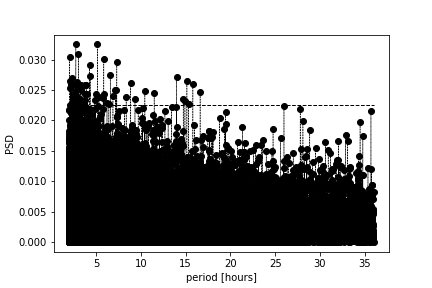 | 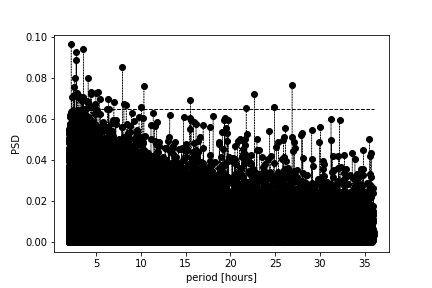 |

**Figure S6: Cosinor regression analysis on the time of labor onset in induced labors.** A – all gestational ages, B – term labors (GA ≥ 37 weeks), C – preterm labors (moderate to late preterm 32 ≤ GA < 37), D – very preterm labors (28 ≤ GA < 32), E – extremely preterm labors (GA < 28), F – very preterm and extremely preterm labors combined (GA < 32). The p-values correspond to the overall significance of each fit.


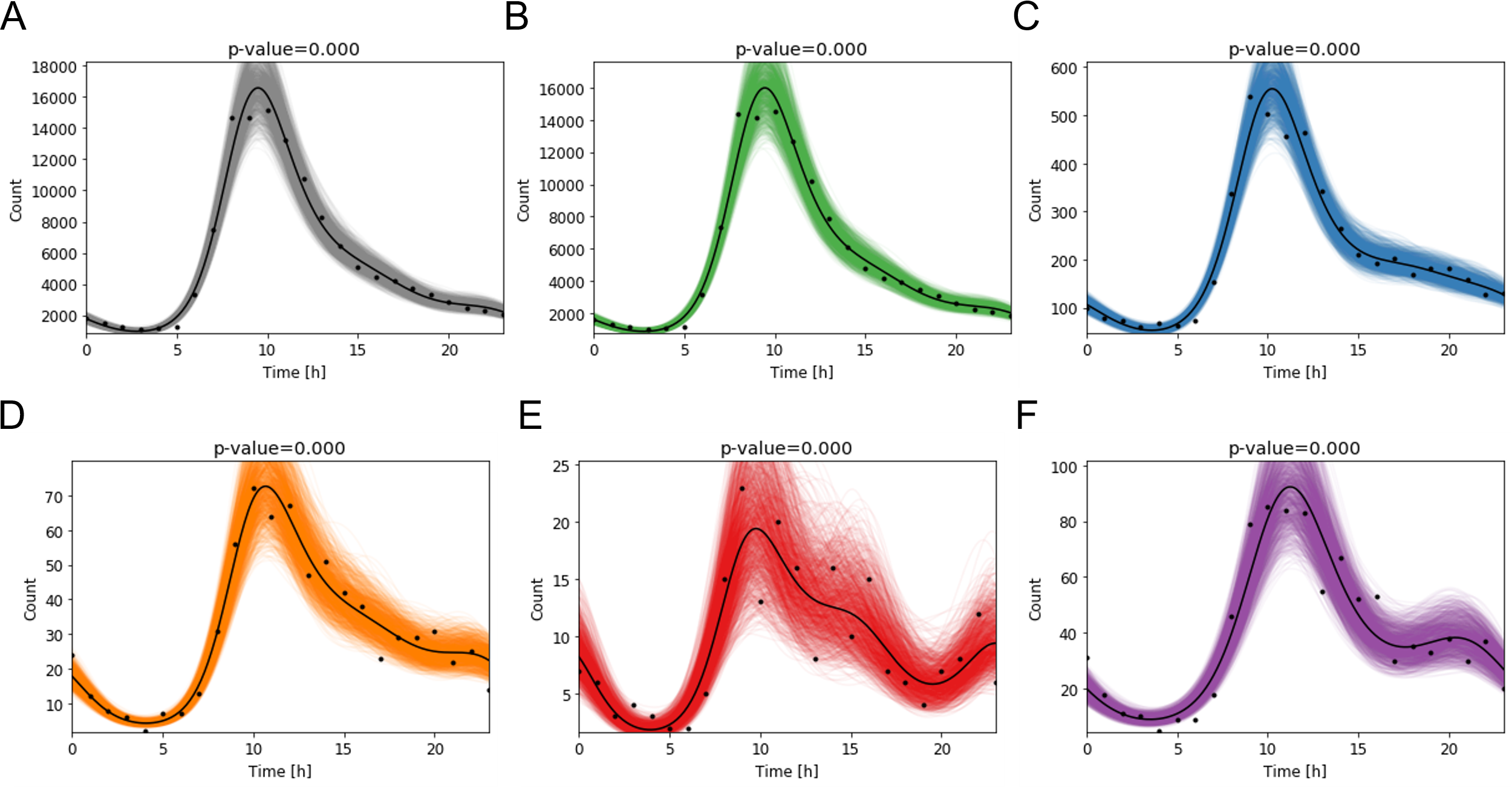


**Figure S7: Cosinor regression performed on the data that correspond to the labors initiated with contractions, whereas each gestational age group is composed of a single week.** The p-values correspond to the overall significance of each fit and n corresponds to the number of cases. w corresponds to the gestational age in weeks.


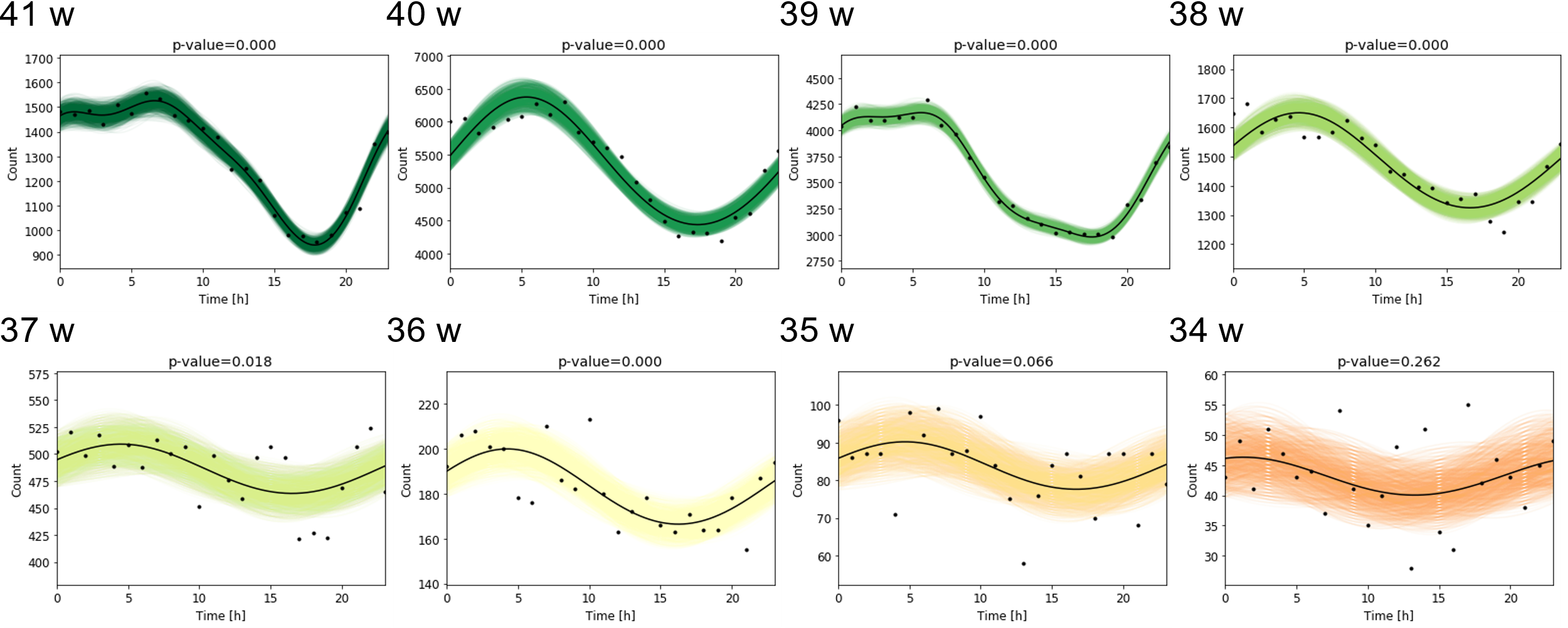


**Figure S8: Cosinor regression performed on the data that correspond to the labors initiated with spontaneous rupture of membranes, whereas each gestational age group is composed of a single week.** The p-values correspond to the overall significance of each fit and n corresponds to the number of cases. w corresponds to the gestational age in weeks.


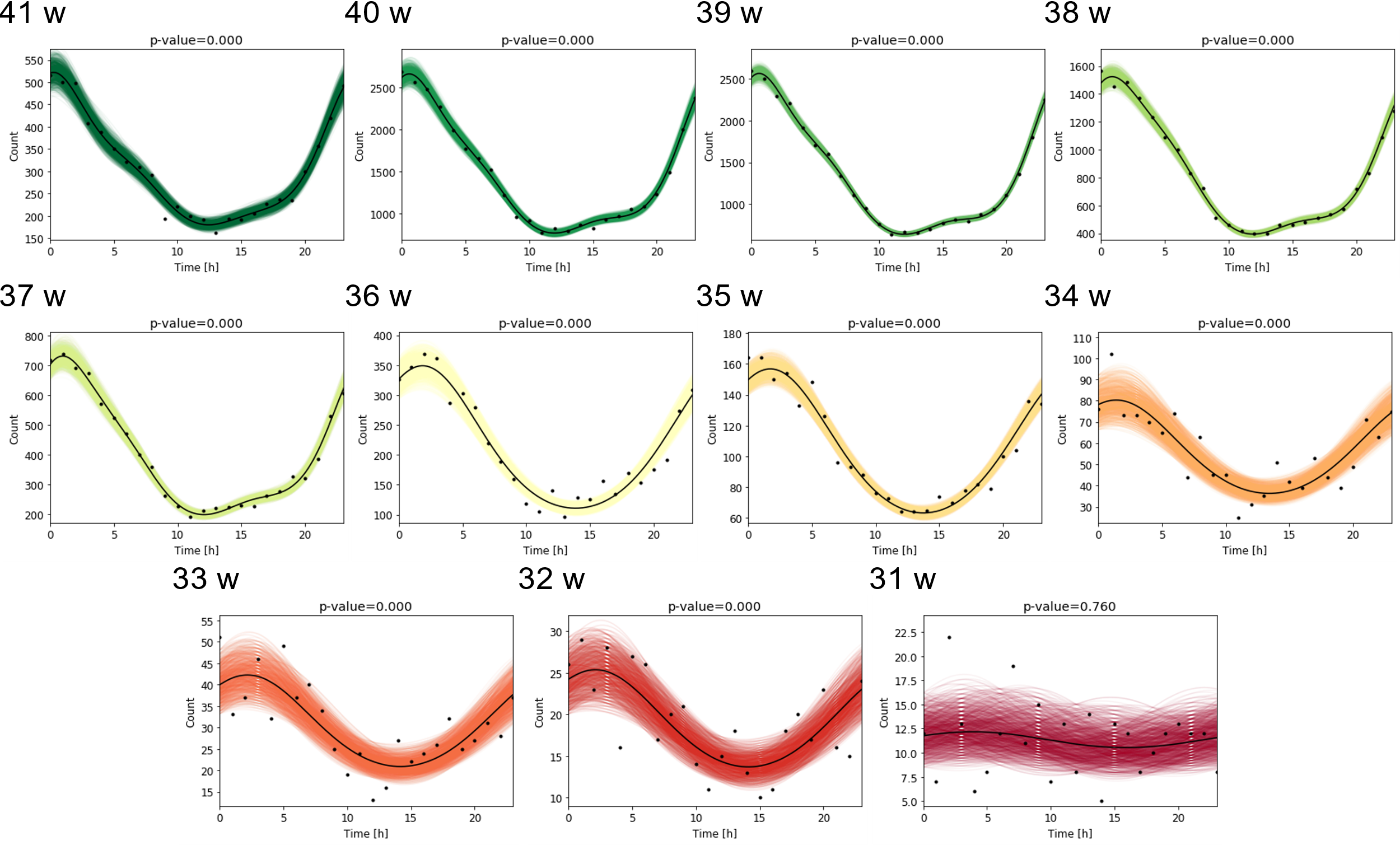

Supplement: Supplementary file 1 — Supplementary Information. [file 41598_2024_54490_MOESM1_ESM.docx]
